# Alkalinity of Neutrophil Phagocytic Vacuoles Is Modulated by HVCN1 and Has Consequences for Myeloperoxidase Activity

**DOI:** 10.1371/journal.pone.0125906

**Published:** 2015-04-17

**Authors:** Adam P. Levine, Michael R. Duchen, Simon de Villiers, Peter R. Rich, Anthony W. Segal

**Affiliations:** 1 Division of Medicine, University College London, London, United Kingdom; 2 Department of Cell and Developmental Biology, University College London, London, United Kingdom; 3 Glynn Laboratory of Bioenergetics, Department of Biology, University College London, London, United Kingdom; University of Alabama-Birmingham, UNITED STATES

## Abstract

The NADPH oxidase of neutrophils, essential for innate immunity, passes electrons across the phagocytic membrane to form superoxide in the phagocytic vacuole. Activity of the oxidase requires that charge movements across the vacuolar membrane are balanced. Using the pH indicator SNARF, we measured changes in pH in the phagocytic vacuole and cytosol of neutrophils. In human cells, the vacuolar pH rose to ~9, and the cytosol acidified slightly. By contrast, in *Hvcn1* knock out mouse neutrophils, the vacuolar pH rose above 11, vacuoles swelled, and the cytosol acidified excessively, demonstrating that ordinarily this channel plays an important role in charge compensation. Proton extrusion was not diminished in *Hvcn1^-/-^* mouse neutrophils arguing against its role in maintaining pH homeostasis across the plasma membrane. Conditions in the vacuole are optimal for bacterial killing by the neutral proteases, cathepsin G and elastase, and not by myeloperoxidase, activity of which was unphysiologically low at alkaline pH.

## Introduction

**Fig 1:**
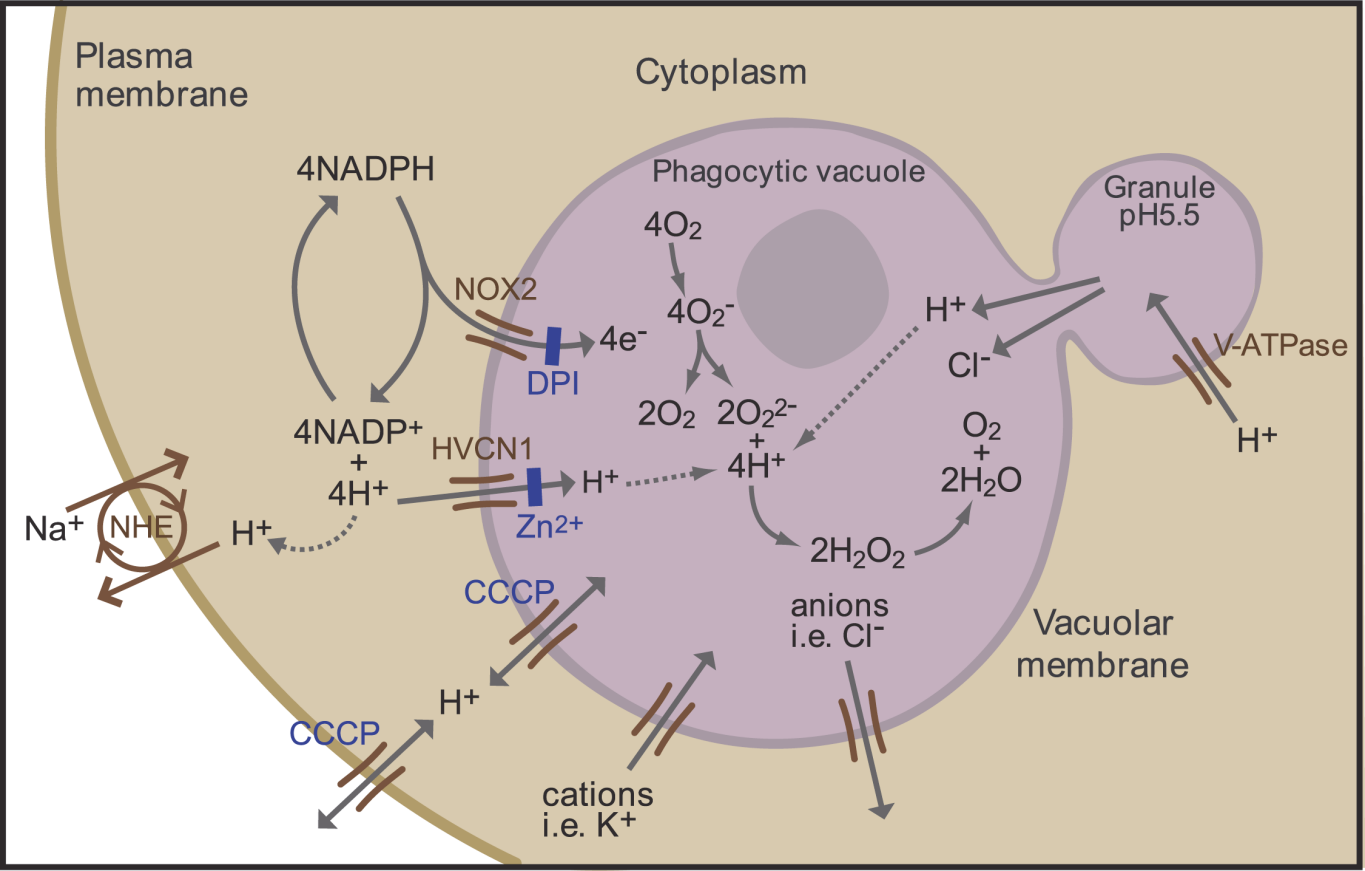
Schematic representation of the neutrophil phagocytic vacuole showing the consequences of electron transport by NOX2 onto oxygen. The proposed ion fluxes that might be required to compensate the movement of charge across the phagocytic membrane together with modulators of ion fluxes are shown.

Neutrophils that encounter a bacterium or fungus engulf it into a phagocytic vacuole of invaginated plasma membrane, into which cytoplasmic granules release their contents of potentially lethal enzymes (Fig 1). These processes are associated with a burst of non-mitochondrial respiration in which electrons are passed across the membrane of the vacuole by an NADPH oxidase, NOX2, that generates superoxide [1]. This electron transport is essential for efficient killing of the microbes as evidenced by the severe immunodeficiency syndrome of Chronic Granulomatous Disease (CGD) in which the function of NOX2 is absent or compromised [2].

The transport of electrons into the phagocytic vacuole is electrogenic, causing a large, rapid, membrane depolarisation which will itself curtail further electron transport unless there is compensatory ion movement [3] by the passage of cations into the vacuole and/or anions in the opposite direction (Fig 1). The nature of the ions that compensate the charge may have a direct effect on the pH within the vacuole and the cytosol. Attempts to characterise mechanisms of charge compensation have concentrated on the role of proton channels [4], characterised using divalent cations such as Zn^2+^ and Cd^2+^ as inhibitors [3,5,6]. These ions are reasonably selective for proton channels in the low micromolar range [4,7], but have multiple other targets when used at millimolar concentrations [8–12]. Cloning of the gene for the proton channel *Hvcn1* [13,14], and the subsequent generation of *Hvcn1* knockout mice [15] has allowed a more precise definition of its role in neutrophil biology. Contrary to predictions from previous studies using high concentrations of Zn^2+^ [8], complete eradication of the HVCN1 channel only reduced oxidase activity by about 50% [15,16], and had a surprisingly small effect on microbial killing [15]. Inhibition or deletion of the HVCN1 channel has been shown to result in exaggerated acidification of the cytosol after phagocytosis of zymosan [17] or stimulation of the oxidase with phorbol myristate acetate (PMA) [18] which led to the suggestion that this channel might be important for the expulsion of protons from the neutrophil cytosol [17,18] although this was not measured directly in either of these studies. Those observations raise the possibility that the depressant effect of the loss of the HVCN1 channel on the NADPH oxidase might be due to the development of an excessively acidic cytosol, which inhibits the oxidase [19], rather than as a consequence of impaired charge compensation.

In a subsequent study of *Hvcn1* ^-/-^ neutrophils phagocytosing zymosan particles [20], averaging the pH of the entire population of neutrophil phagosomes did not yield a significant difference between wild type and *Hvcn1*-deficient cells. However, the pH of *Hvcn1* ^-/-^ phagosomes were found to be heterogeneous, with a third being alkaline and 14% acidic.

In order to define the role of the HVCN1 channel in neutrophil biology, we have used the ratiometric fluorescent pH indicator, SNARF, to measure the pH directly in individual phagocytic vacuoles and in the cytosol of human, and wild type (WT) and *Hvcn1* ^-/-^ mouse neutrophils during phagocytosis. The data confirm that the HVCN1 channel plays a major role in charge compensation across the phagocytic vacuole membrane, and in its absence non-proton fluxes predominate in charge compensation.

There are two opposing views as to the mechanisms by which the oxidase contributes to microbial killing. On the one hand it is believed that myeloperoxidase (MPO) promotes killing through the peroxidatic generation of hypochlorous acid from H_2_O_2_ produced by the oxidase [21]. An alternative view is that killing is mediated through the digestive action of neutral proteases that are activated by the elevated pH [22] and influx of K^+^ [23] generated by the oxidase. To determine which of these mechanisms is the more feasible within the environment of the phagocytic vacuole, we measured the pH dependence of the activity of MPO and that of the two major neutral proteases, cathepsin G and elastase, and related these to the observed phagocytic vacuolar pH in normal neutrophils.

## Results

### The respiratory burst is impaired in *Hvcn1* ^-/-^ neutrophils whilst extracellular acid release is normal

**Fig 2:**
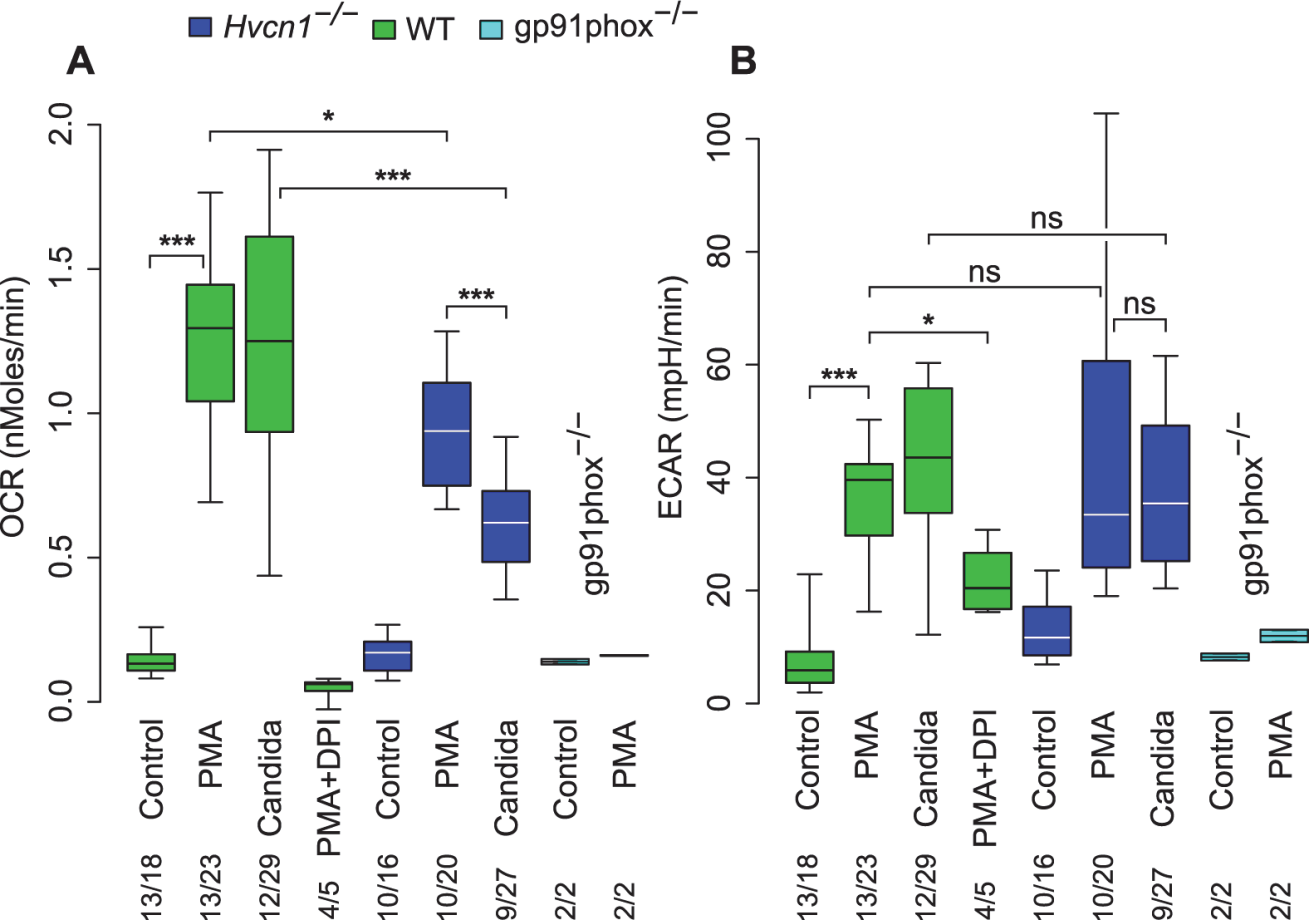
Oxygen consumption and extracellular acidification rate by neutrophils. (A) Oxygen consumption rate (OCR) and (B) extracellular acidification rate (ECAR) by neutrophils from WT, *Hvcn1* ^-/-^ and gp91^phox-/-^ mice in response to stimulation with PMA (with and without DPI) or opsonised *Candida*. The numbers of independent experiments is shown over the total number of measurements. Statistical significance: *** p<0.001, ** p<0.01 and * p<0.05. Differences between PMA stimulated WT and gp91^phox-/-^ were p<0.001 in (A) and p<0.05 in (B). There were no significant differences in (B) between PMA and *Candida* or between WT and *Hvcn1* ^-/-^ cells. Median, quartiles and 95% centiles are shown.

In WT neutrophils, oxygen consumption was increased tenfold by PMA and to a similar level by the addition of opsonised *Candida*. PMA stimulated oxygen consumption was reduced to 73% of normal in cells isolated from *Hvcn1* ^-/-^ mice (p = 0.035), consistent with previous observations [15,16,18] was abolished by the oxidase inhibitor DPI (p<0.001), and was absent in neutrophils lacking gp91^phox^ (p<0.001) (Fig 2A). Oxygen consumption by *Hvcn1* ^-/-^ cells phagocytosing opsonised *Candida* was 50% of that by WT mouse neutrophils (p<0.001), and significantly lower than in the same cells after PMA stimulation (p<0.001) but higher than the 20% measured by others [20]. Using Amplex Red to measure H_2_O_2_ generated by PMA stimulated cells, oxidase activity was decreased to similar levels in *Hvcn1* ^-/-^ at 58.4% ± 18.1 (SD) (n = 5) of that by WT cells.

Activation of the oxidase using PMA or *Candida* increased the extracellular acidification rate (ECAR), to similar levels in *Hvcn1* ^-/-^ and WT mouse neutrophils and levels achieved were equivalent with PMA or with *Candida* (p = ns for all comparisons). The rate was significantly reduced in the presence of DPI (p = 0.035) and remained at baseline levels in neutrophils lacking gp91^phox^ (p<0.01) (Fig 2B). The relatively high extracellular acidification observed when oxygen consumption by PMA stimulated cells is blocked by DPI can be explained by the inhibitory effect of DPI on mitochondrial electron transport [24] as well as that of the neutrophil oxidase. Inhibition of mitochondrial electron transport leads to anaerobic glycolysis with the production of lactate and the release of more acid [25]. No difference was observed between the extracellular acidification between WT and *Hvcn1* ^-/-^ cells in the presence of PMA and DPI (data not shown).

Oxygen consumption by human neutrophils phagocytosing *Candida*, as measured with a Clark oxygen electrode, was determined as 5.8 fmols ± 0.4 (SD) (n = 5) per *Candida* phagocytosed and the respiratory burst was brief, with a peak rate lasting about two minutes [26]. In neutrophils from the *Hvcn1* ^-/-^ mice the equivalent measurement was 3.2 fmols corresponding to the ~50% reduction observed (Fig 2A). Given the 4:1 ratio of electrons passing across the vacuolar membrane to oxygen consumed (Fig 1), the consumption of ~6 fmols of oxygen per *Candida* would require ~24 fmols of compensating charge in normal neutrophils and ~12 fmols in *Hvcn1* ^-/-^ cells.

### The phagocytic vacuole of human and WT mouse neutrophils reaches pH 8.5–9 whilst in *Hvcn1* ^-/-^ cells it exceeds 11

**Fig 3:**
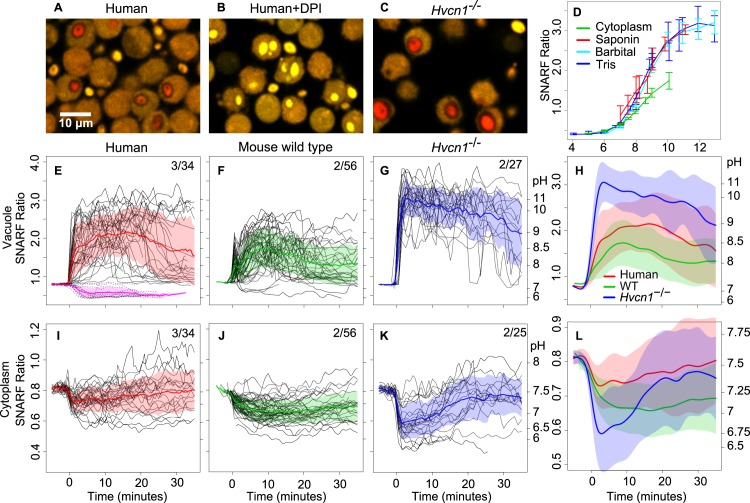
Time courses of changes in pH in the vacuole and cytoplasm of phagocytosing neutrophils. Representative images of *Candida* phagocytosed by human neutrophils (A), with DPI (B), and by *Hvcn1* ^*-/-*^ neutrophils (C). Standard curves for the relationship between SNARF ratio and pH of organisms and cytoplasm are shown in (D). *Candida* alone were added to two different buffer systems, labelled Tris or Barbital, and intracellular organisms were exposed to the Barbital buffers after permeabilisation of neutrophils with saponin. Panels E-L show time courses of the pH changes of phagocytosed *Candida* and cytoplasm of human (E, I), mouse WT (F, J) and *Hvcn1* ^-/-^ (G, K) neutrophils synchronised to the time of particle uptake (0 minutes). In E-G and I-K, each individual black line represents serial measurements of the SNARF ratio of a single phagocytosed *Candida* or neutrophil cytoplasm, respectively. Mean ± SD (shaded areas) are shown. In the composite panels H and L the mean data have been smoothed. Data are plotted according to SNARF ratio with the approximate corresponding pH shown on the right y-axis. The number of independent experiments over the total number of individual cells examined is shown. The effect of DPI on vacuolar pH in human neutrophils is shown in E (pink and dashed lines, 12 cells).

**Fig 4:**
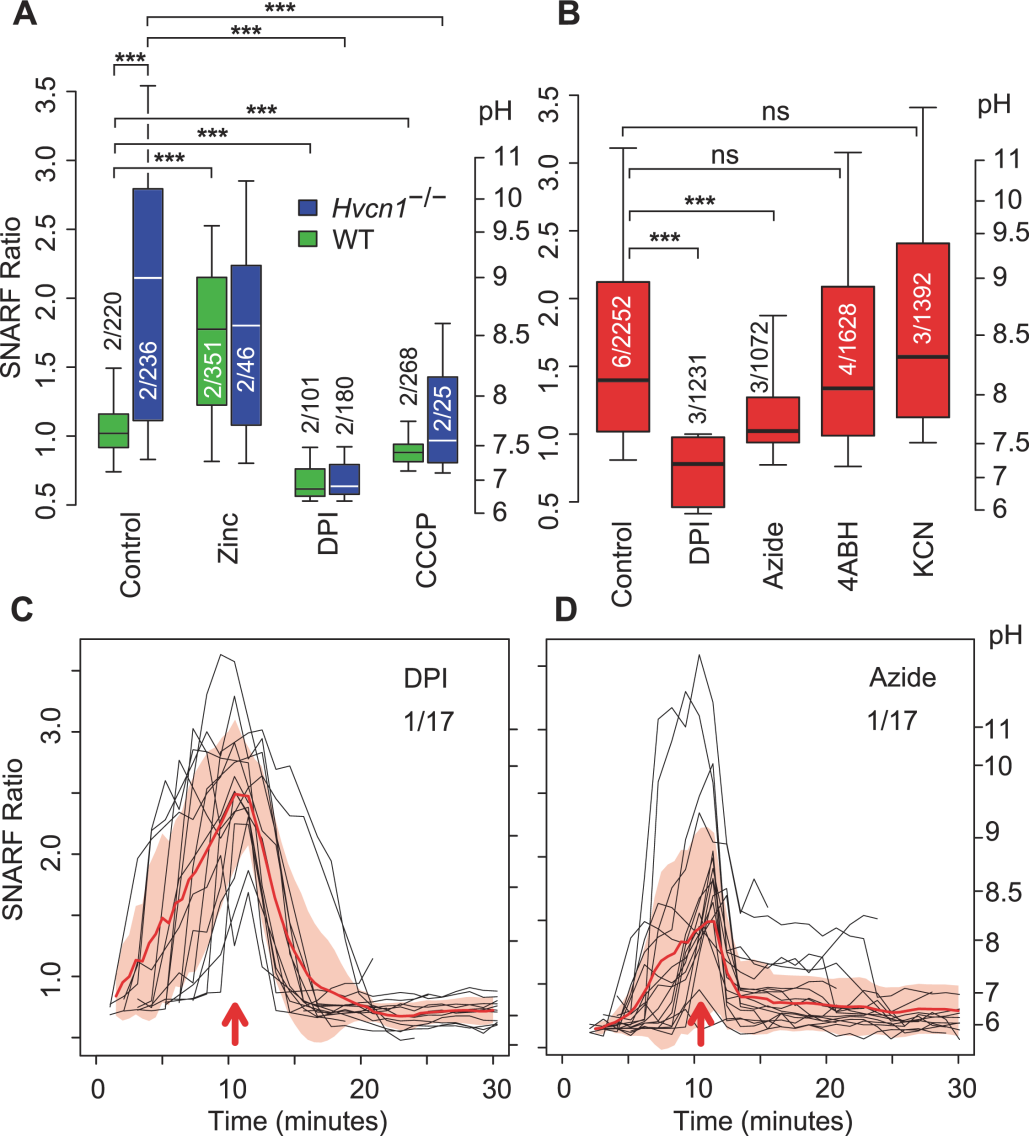
The effect of DPI, azide, 4ABH, KCN, zinc, and CCCP on vacuolar pH. (A) The effect of 300 μM Zn^2+^, DPI and 60 μM CCCP on vacuolar pH in WT and *Hvcn1* ^-/-^ neutrophils at ~30 minutes following the addition of *Candida* without synchronisation to particle uptake. (B) The effect of 5 μM DPI, 5 mM sodium azide, 50 μM 4ABH and 1mM KCN, on vacuolar pH in human neutrophils at ~30 minutes following the addition of *Candida* without synchronisation to particle uptake. The effect of the addition of azide (C) or DPI (D) on vacuolar pH in human neutrophils synchronised to time of addition (red arrows). The numbers of independent experiments is shown over the total number of measurements. Median, quartiles and 95% centiles are shown. Statistical significance: *** p<0.001.

The fluorescence ratio of SNARF coupled to heat killed *Candida* showed a sigmoidal relationship with pH and was approximately linear with pH 7–10 (Fig 3D). We tracked changes in pH in the phagocytic vacuoles of individual neutrophils from the time that labelled *Candida* were engulfed. Shortly following engulfment, the vacuole underwent a significant alkalinisation (Fig 3). In human neutrophils, this started almost immediately (Fig 3E and S1 Video). The mean maximum pH reached post-phagocytosis was 9.0 (SD 8.3–10.2) (Fig 3E) and this elevated pH was maintained for 20–30 minutes. When the NADPH oxidase was inhibited by DPI, the vacuole acidified to about 6.3 (SD 6.1–6.56) (Fig 3B and 3E), although SNARF determinations are not very accurate at this pH, these results are similar to previous findings [27] and when added following phagocytosis, DPI rapidly reduced the vacuolar pH (Fig 4C and S2 Video) [20]. These elevations in vacuolar pH occurred despite the buffering capacity of the heat-killed *Candida* (S1 Fig). The maximum difference in the vacuolar pH in human neutrophils in the presence or absence of DPI (~6.5 and ~9, respectively (Fig 3E)) required 1.9 fmols of OH^-^ per *Candida*. Assuming this pH rise was entirely due to non-proton charge compensation, it would amount to ~8% of the total ~24 fmol compensating charge, close to the ~5% previously estimated [23].

WT mouse neutrophils showed a similar, although less extreme, alkalinisation of the vacuole reaching a mean maximum pH of ~8.5 (SD 8.0–9.1) post-phagocytosis (Fig 3F). The initial rise was very rapid and was followed by a brief fall, possibly as a result of degranulation of acid granule contents, before resuming its upward course after a delay of about one minute. By contrast, the pH in the vacuoles of *Hvcn1* ^-/-^ neutrophils was rapidly and grossly elevated to a mean value of ~11.1 (SD 9.7–12.5) (Fig 3G and S3 Video). These changes were observed in almost all vacuoles. The difference between the areas under the curves of the WT and *Hvcn1* ^-/-^ cells was highly significant (p<0.001). Given the strong buffering capacity of *Candida* in the alkaline range, a rise in pH from ~6.5 in DPI treated cells to ~11.1 in *Hvcn1* ^-/-^ required ~9.2 fmols of OH^-^ per organism (S1 Fig), similar to the ~12 fmols of total compensating charge. In a small number of *Hvcn1* ^-/-^ cells (3.3 ± 3.8 SD of 8 experiments and 810 cells) the vacuoles collapsed (e.g. S3 Video and S4 Video), which coincided with sudden alkalinisation of the cytoplasm. These extreme conditions could impair the oxidase in phagocytosing neutrophils.

The alkalinisation in *Hvcn1* ^-/-^ vacuoles was almost completely prevented by the addition of the protonophore carbonyl cyanide-m-chlorophenylhydrazone (CCCP), which will produce a proton shunt, whereas Zn^2+^, increased alkalinisation of vacuoles in WT mouse neutrophils at a concentration of 100 μM (Fig 4A). Sodium azide has been used [20,28] to inhibit the action of MPO which was thought to quench the fluorescence of fluorescein, the flurophore previously utilised to determine vacuolar pH. We therefore determined the effect of the addition of azide on SNARF fluorescence. In endpoint assays, azide significantly reduced the pH in the vacuoles at ~30 minutes in human neutrophils (p<0.001, Fig 4B) or almost immediately when added during a time course (Fig 4D). This effect of azide was not due to its inhibitory effect on MPO because neither 4-aminobenzoic acid hydrazide (4ABH), a selective inhibitor of MPO, at 50 μM, more than three times the IC50 [29] (Fig 4B), nor 1mM KCN, depressed vacuolar pH. KCN showed complete saturation of binding to MPO in intact neutrophils at this concentration (S2 Fig) at which it inhibits its peroxidatic activity [30]. When SNARF-labelled *Candida* were added to buffers at varying pH in the absence or presence of azide, no effect of azide was seen on the SNARF ratio (S3 Fig) of the *Candida* themselves.

### Cytoplasmic pH falls after phagocytosis, and this is exaggerated in *Hvcn1* ^-/-^ cells

In human or WT mouse neutrophils the respiratory burst was associated with a modest acidification of the cytoplasm, from a pH of 7.56 (SD 7.49–7.62) (S4D Fig) to a mean nadir pH of 7.30 (SD 7.09–7.49) (Fig 3I and S1 Video), with homeostasis being achieved over the ensuing 20–30 minutes as described previously [31–33]. Neutrophils from WT mice behaved similarly (Fig 3J). In *Hvcn1* ^-/-^ neutrophils the acidification was much more pronounced (p<0.001, Fig 3K and S3 Video), reaching a minimum mean pH of ~6.72 (SD 6.08–7.12) at 2.0 minutes after phagocytosis, before returning to normal after about 20–30 minutes. The rate of recovery of cytoplasmic pH after acidification with 20 mM NH_4_Cl for five and 20 minutes was no different between WT and *Hvcn1* ^-/-^ neutrophils (S4A and S4B Fig, respectively).

The buffering capacity of the neutrophil cytosol has previously been determined at between 28 [34] and 50 [32] mM unit^-1^. Given an approximate neutrophil volume of ~300 μm^3^ [35] and the 0.2 unit fall in cytoplasmic pH in human neutrophils (from 7.5 to 7.3) this equates to the accumulation of ~2.3 fmols (39 mM unit^-1^ × 0.2 unit × 300 μm^3^) of protons left in the cytosol when the charge is compensated by alternative ions (9.6% of ~24 fmol). In *Hvcn1* ^-/-^ cells the fall in cytoplasmic pH to 6.6 equates to ~10.5 fmol of protons (calculated as above).

### *Hvcn1* ^-/-^ vacuoles are larger than normal

**Fig 5:**
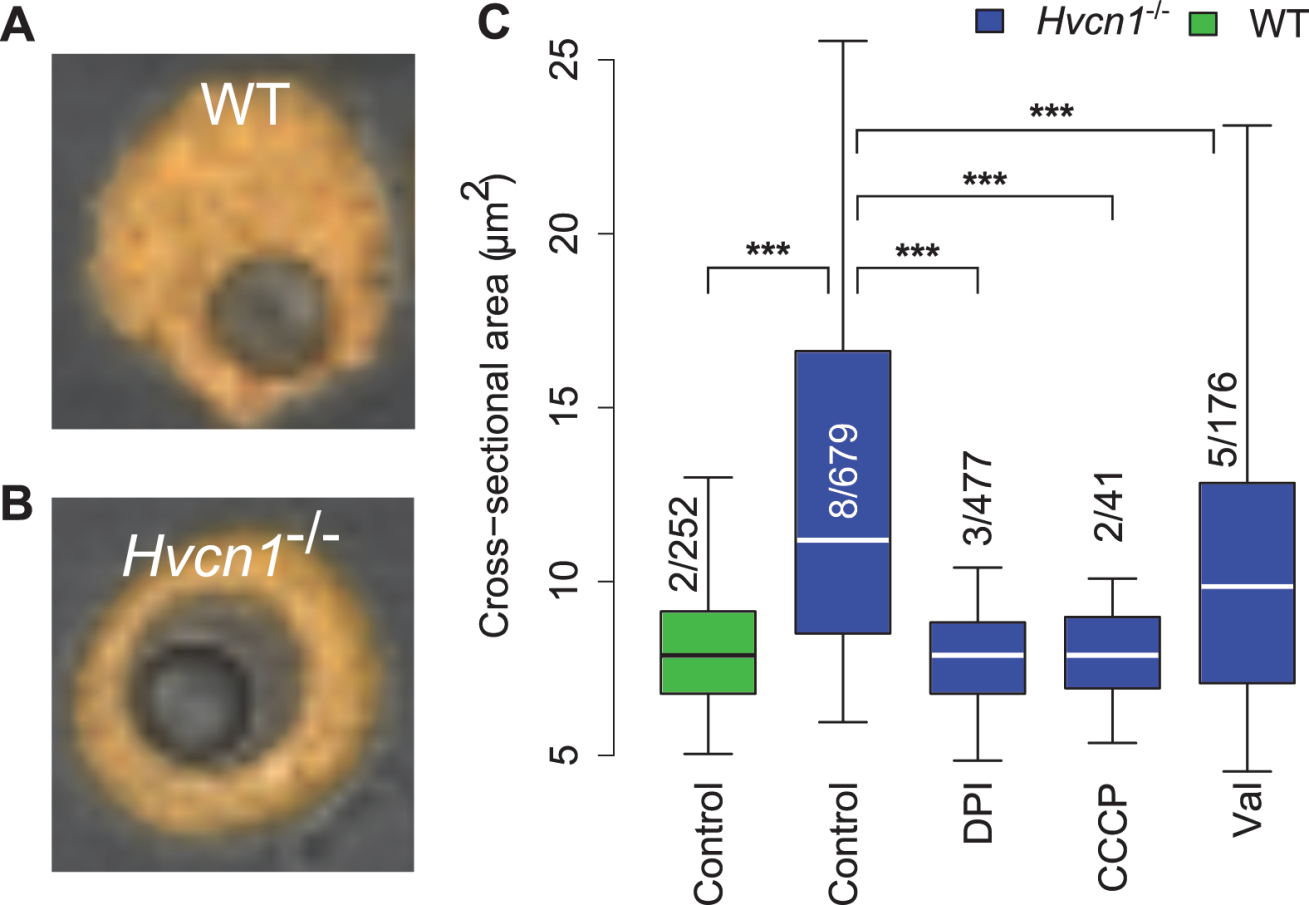
Vacuolar size in WT and *Hvcn1* ^-/-^ neutrophils containing a single latex particle. The cross-sectional area of a latex particle is ~7 μm^2^. Representative images of a WT (A) and *Hvcn1* ^*-/-*^ neutrophil containing a single latex particle are shown in (A) and (B), respectively. (C) Quantitation of vacuolar swelling in *Hvcn1* ^-/-^ neutrophils compared with WT, and the effects of 5 μM DPI, 60 μM CCCP and 3 μM valinomycin. The numbers of independent experiments is shown over the total number of measurements. Median, quartiles and 95% centiles are shown. Statistical significance: *** p *<* 0.001 and ** p<0.01.

The vacuoles of *Hvcn1* ^-/-^ neutrophils containing *Candida* underwent a profound swelling, which was much more obvious than in WT mouse or human cells (S1 Video and S3 Video). To determine the extent of this swelling and to distinguish it from that induced by the osmotic effects of the products of digestion of the *Candida*, we measured the cross-sectional areas of vacuoles containing a single indigestible latex particle with a diameter of 3 μm (a cross-sectional area of 7.1 μm^2^), similar to that of *Candida*. In neutrophils from *Hvcn1* ^-/-^ mice, the vacuoles swelled to a median cross-sectional area of 11.2 μm^2^ (quartiles 8.5–16.6) compared with 7.9 μm^2^ (6.8–9.1) in neutrophils from WT mice (p<0.001) or *Hvcn1* ^-/-^ treated with 5 μM DPI (7.9 μm^2^) (6.8–8.8) or CCCP (7.9 μm^2^) (6.9–9.0) (Fig 5C) after 30 minutes. These results indicate that osmotically active ions are driven into the vacuole by the oxidase in the absence of the proton channel. This swelling was reduced significantly (p<0.001) by 3 μM valinomycin to a median area of 9.9 μm^2^ (7.1–12.8). The estimated volumes utilising the radius calculated from the median cross-sectional area and assuming spherical vacuoles were: latex particles (14.2 μm^3^); WT vacuoles (16.7 μm^3^) and *Hvcn1* ^-/-^ (28.2 μm^3^) without and (23.4 μm^3^) with valinomycin, respectively.

If the charge compensation in *Hvcn1* ^-/-^ cells (~12 fmols) were purely accomplished by the influx of cations, e.g. K^+^, to a concentration that balanced the cytosolic osmotic pressure of ~300 mOsm, taken as close to that of extracellular fluid [36], it would produce a vacuolar volume of ~40 μm^3^ (12 fmols / 300 mM) greater than that of the latex particles alone, considerably larger than that observed. This suggests that non-osmotically active cations, such as protons, pass into the vacuole, or that anions such as Cl^-^, pass out, or a combination of these two events.

### Physiological pH in human neutrophil vacuoles is optimal for activity of granule enzymes cathepsin G and neutrophil elastase but incompatible with effective peroxidatic and chlorinating function of MPO

**Fig 6:**
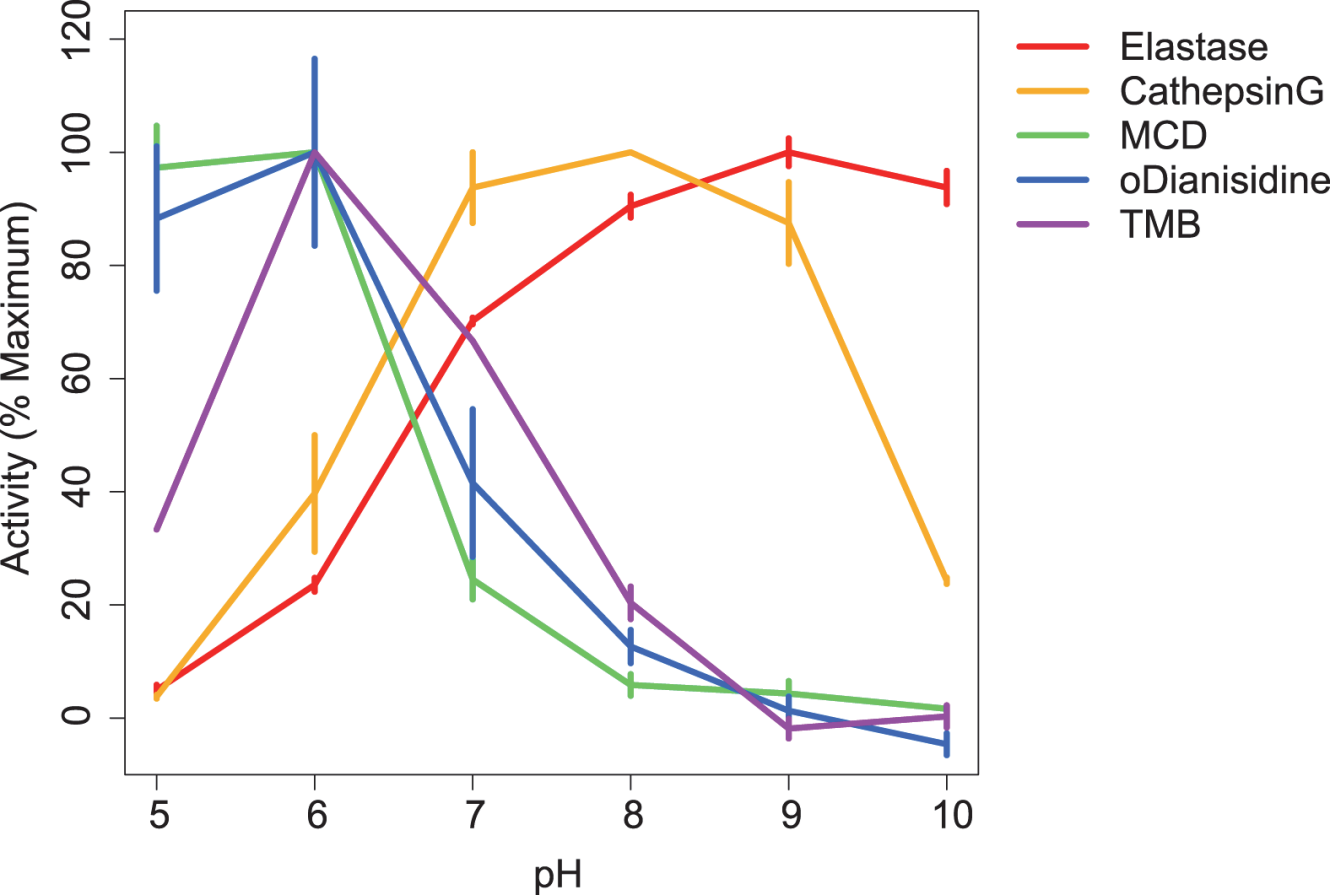
Effects of pH on enzymatic activity. The effect of variations in pH on peroxidatic and chlorinating activities of MPO and on the protease activities of cathepsin G and of elastase are shown. Results shown are the mean + SD of at least three separate assays and are expressed as a percentage of the maximal observed activity.

The influence of pH on the peroxidatic and chlorinating activities of MPO and on the protease activities of cathepsin G and elastase are shown in Fig 6. Peroxidase and chlorinating activities of MPO were maximal at acid pH, with kcat for TMB oxidation and MCD chlorination s^-1^ at pH 5.0 being 36 and 19, respectively. Both activities fell off substantially with elevated pH until virtually nothing of either could be detected at pH 9 or 10. By contrast, activity of both cathepsin G and elastase was low at pH 5, and that for cathepsin G peaked at between 7 and 9 whereas elastase was most active between 8 and 10.

The SOD activity of MPO showed a similar relationship to pH as did its peroxidase activity whereas the catalatic activity was lowest at neutral pH and increased at pH 5 and 6, and 10 at which kcat = 3 O_2_ generated s^-1^ (S5 Fig).

## Discussion

The original description [22] of an alkalinisation of the neutrophil phagocytic vacuole by the NADPH oxidase to a pH of ~7.5–8 has been replicated a number of times [27,37,38]. In those studies, fluorescein was employed as pH indicator. Fluorescein saturates at a pH of ~8 and above, was not generally used in a ratiometric manner. In addition, it has been thought by some to become bleached by the action of MPO in the phagocytic vacuole [39,40].

SNARF is much better suited to the determination of the vacuolar pH. It has a dynamic range of between pH 6 and 11 with approximate linearity between 7 and 10 (Fig 3D), is ratiometric, and can also be used to simultaneously measure the pH in the vacuole and cytoplasm. Outside of pH interval, SNARF-inferred pH measurements are less precise. We have shown in several ways that its fluorescent properties are not altered by the conditions in the phagocytic vacuole. The extent and duration of alkalinisation we observed in the human neutrophils was much greater than that previously described by us [22] and others, in part because SNARF is more suited to measurements in the alkaline range. In addition, we only measured the pH of *Candida* inside vacuoles, so that the fluorescence signal was not influenced by that of the non-phagocytosed organisms that are buffered by the extracellular medium. Synchronising the changes in fluorescence to the time of particle uptake, gave a more accurate indication of the changes in pH over time.

Both studies that were unable to detect an elevation of neutrophil vacuolar pH above neutral [20,28] included sodium azide in all the solutions to counteract bleaching of the fluorescein by MPO [28]. We found here that azide significantly reduced the vacuolar pH (Fig 4B and 4D) by a mechanism that did not involve the inhibition of MPO; 4ABH, which is a more specific MPO inhibitor, had no such effect at a concentration of over three times the IC50 [29] and the addition of azide produced a rapid drop in the pH of previously alkaline vacuoles (Fig 4D). KCN, which inhibits MPO [41], also had no effect on vacuolar pH at concentrations at which we demonstrated it to bind to MPO to saturation in intact neutrophils. El Chemaly and colleagues [20] observed three different populations of phagocytic vacuole in *Hvcn1* ^-/-^ neutrophils, a third reaching a pH of over 8.3, surprisingly high when using fluorescein as indicator [27], and 14% were acidic. They correctly concluded that the alkaline vacuoles indicated that the HVCN1 channel transported protons into the vacuole. They suggested that the oxidase repelled V-ATPases from association with the vacuoles, and that the acidic vacuoles were caused by weak oxidase repulsion of these V-ATPases which then pumped protons into the vacuole. We did not observe the same heterogeneity with our cells which were purified from peripheral blood (Fig 3E). We also observed a very alkaline and a more acidic population of vacuoles when we performed experiments on bone marrow (data not shown). It is possible that some immature neutrophils in the bone marrow have an abnormally low respiratory burst.

The accurate measurement of vacuolar pH in normal human neutrophils is absolutely central to understanding the mechanisms by which these cells kill bacteria and fungi. A strong body of opinion supports the concept that MPO is “a front line defender against phagocytosed microorganisms” [21] and that it kills microbes within this compartment through the generation of HOCl. The virtual absence of peroxidase and chlorinating activity of MPO at pH of between 8.5 and 9.5 pertaining in the vacuole, and the demonstration that the antimicrobial activity of MPO is only observed under acid conditions [41], argues strongly against this possibility. However, as shown here, these pH provide an optimal milieu for the microbicidal and digestive functions of the major granule proteases, elastase and cathepsin G which are activated by this elevated pH and the influx of K^+^ into the vacuole [23,42]. These results support the finding that knock-out mice lacking cathepsin G and elastase, whilst retaining normal oxidase and MPO activities, are rendered unduly susceptible to infection by *Staphylococcus aureus*, *Candida albicans* and *Aspergillus fumigatus* [23,43,44]. It is possible that MPO has two different functions *in vivo*, depending upon the local environment. The pH of ~9 in the vacuole favours catalase activity rather that of a peroxidase, whereas, when the neutrophil encounters an organism that it is unable to fully engulf, such as a fungal mycelium, the situation is different [45]. The concentration of MPO and H_2_O_2_ will be much lower, the pH, which is low in inflammatory foci at ~6 [46] is optimal for peroxidatic activity, and the supply of chloride is limitless.

The gross difference in the vacuolar pH between *Hvcn1* ^-/-^ neutrophils and those from WT mice and humans must indicate that fewer protons pass into the vacuole in the *Hvcn1* ^-/-^ cells, providing firm evidence that under physiological conditions the HVCN1 channel compensates the oxidase induced charge by conducting protons across the vacuolar membrane. The excessive acidity that develops in the cytoplasm in *Hvcn1* ^-/-^ neutrophils, despite a normal proton extrusion rate out of the cell, is further evidence that the protons generated by activity of the oxidase on the cytosolic side of the vacuolar membrane accumulate because they do not pass into the vacuole. There was no difference in proton extrusion from *Hvcn1* ^-/-^ and WT neutrophils despite approximately half the oxygen consumption by the former which can be explained as follows. The transfer of one electron across the vacuolar membrane by the NADPH oxidase produces two protons in the cytoplasm, one by the production of NADP from NADPH, and the other when the NADPH is regenerated by the hexose monophosphate shunt [47]. In WT cells, approximately one half of these protons pass into the vacuole through the HVCN1 channel to compensate the charge and the remainder are expelled to the exterior whereas in the *Hvcn1* ^-/-^ cells the protons that would normally pass into the vacuole and those that would normally be released into the extracellular medium are both expelled. Additional proof that it is the failure of the movement of protons into the vacuole that causes the extreme alkalinisation in the *Hvcn1* ^-/-^phagocytic vacuoles is provided by the effect of the addition of the protonophore, CCCP, which overcomes the barrier to proton flux and largely reverses the alkalinisation. These results provide direct evidence for compensation by the HVCN1 channel of the NADPH oxidase induced charge across the vacuolar membrane.

Two studies have concluded that the HVCN1 channel plays an important role in controlling the cytoplasmic pH by extruding protons into the extracellular medium. The first [18] used 2,7-bis-(carboxyethyl)-5-(and-6)-carboxyfluorescein (BCECF) as indicator in *Hvcn1* ^-/-^ cells which had been suspended in a sodium free medium to prevent the activity of Na^+^/H^+^ exchangers [48,49] which are known to regulate cytoplasmic pH. The cells were stimulated with PMA, which leads to activation of the oxidase at the plasma membrane, where both charge compensation and restitution of the pH should occur. The second study [17] measured cytosolic pH with SNARF in human neutrophils phagocytosing zymosan in the presence of 100 μM Zn^2+^. In these experiments it was uncertain what effect the Zn^2+^, and/or the 10% ethanol in which the cells were bathed for 15 minutes, might be having on cellular processes other than proton transport through the HVCN1 channel. *Hvcn1* ^-/-^ cells recovered from frozen bone marrow were also studied, but the recovered ‘phagocytes’ would have been predominantly macrophages as neutrophils are very vulnerable to freezing [50].

In neither of those studies was the rate of excretion of protons into the extracellular medium measured. In the present study we have shown that proton extrusion rates by *Hvcn1* ^-/-^ cells are not detectably different from those of wild type cells. The rate of proton extrusion is limited in normal cells as shown by the initial fall and relatively slow recovery of cytoplasmic pH in phagocytosing cells. With this as a rate limiting step the fall in cytoplasmic pH, will be directly dependent upon the rate at which protons are generated. Consequently, with rates of proton extrusion equal to those of normal cells, the excessive acidification of the cytoplasm observed in *Hvcn1* ^-/-^ cells can be explained by the failure of the majority of protons generated by the oxidase to move into the vacuole.

There was surprisingly good agreement between the observed oxygen consumption and pH changes and the calculations of the stoichiometry of the required compensating charges and the proportions of these contributed to by protons, 5–10% in normal cells and *>*90% in *Hvcn1* ^-/-^. However, the vacuolar swelling observed was less than would be expected were all the non-proton mediated charge compensation to be due to the influx of K^+^, indicating that part of the charge compensation in these cells may be due to the efflux of anions, e.g. Cl^-^, from the vacuole. A role for K^+^ influx [23] is supported by our observations of the reduction in vacuole swelling by valinomycin and previous data on Rb^+^ efflux from PMA stimulated neutrophils [51,52].

Cytoplasmic granule acidification requires the vacuolar-type H^+^-ATPase to pump protons into the lumen of the granules [53] and a counter-ion flux would be required to neutralize the membrane potential created by proton accumulation. Cl^-^ is the likely counter ion [54], which could enter the granules through CLIC1 [55]. Granule fusion might deliver chloride ions into vacuoles and these ions could subsequently provide charge compensation by passing chloride from the vacuolar lumen to the cytosol through vacuolar chloride channels. The Cl^-^ in the granules would not provide sufficient non-proton charge compensation by itself. A granule volume of about 10% of that of the neutrophil, with a Cl^-^ concentration of ~80 mM [56], and the degranulation of a quarter of the granules into each vacuole, would only produce 1 fmol, or 10% of the required compensating charge in *Hvcn1* ^-/-^ neutrophils, so some mechanism would be required to regenerate Cl^-^ in the vacuole. Chloride movement into phagocytic vacuoles has been proposed to occur through CFTR [57] and CLCN3 [58], but this would require movement against a strong electrical gradient. KCC3 has been shown to be required for normal oxidase activity [51] and being an electroneutral co-transporter of K^+^ and Cl^-^, it could satisfy the dual requirements of moving chloride into the vacuole to be cycled out through a chloride channel whilst leaving K^+^ in the vacuole to activate the granule enzymes [23].

## Materials and Methods

### Materials

SNARF-1 was from Invitrogen. Other chemicals, and latex particles, were from Sigma.

### Media

Balanced salt solution (BSS) contained 156 mM NaCl, 3.0 mM KCl, 1.25mM KH_2_PO^4^, 2 mM MgSO_4_, 2 mM CaCl_2_, 10 mM glucose, 10mM Hepes at pH 7.4. The KH_2_PO^4^ was replaced with KCl in experiments employing Zn^2+^. Phosphate buffered saline (PBS) was from Gibco.

### Ethical approval

All animal work was conducted with the licence and approval of the United Kingdom Home Office. Human participation in this research was approved by the Joint UCL/UCLH Committees on the Ethics of Human Research. All participants provided informed consent.

### Organisms

*Candida albicans* was a clinical isolate.

### Labelling *Candida* with SNARF

*Candida* were washed twice, resuspended in PBS, heated to 60°C for 30 minutes and washed and resuspended at 1×10^8^/ml in 0.1 M NaHCO_3_ pH 8.5. 50 μg SNARF-1 succinimdyl ester in 100 μl dimethyl sulphoxide (DMSO) was added drop wise to 2 ml of a rapidly stirred suspension of these cells at room temperature. The heat killed *Candida* were permeabilised and the SNARF labelled internal structures. Only a thin layer of label was seen on the surface of intact *Candida*. After mixing for 30 minutes at room temperature, the labelled cells were washed twice, and then resuspended in 2 ml of BSS.

### Particle opsonisation

C57B6 mice were injected three times over 6 weeks with 1×10^7^ heat killed (60°C, 30 minutes) *Candida*. 1×10^7^ SNARF labelled *Candida*, in 100μl PBS were incubated with 10% complement preserved serum (Patricell) and 50 μl immune serum for 60 minutes at 37°C. Latex particles 3 μm (Sigma) in 0.1 M NaHCO3 pH 8.5 were opsonised with an equal volume of normal mouse IgG (Caltag) overnight at 4°C. For studies on human neutrophils, *Candida* were opsonised as above with 50% human IgG (160 mg/ml Vivaglobulin CSL Behring) plus 10% pooled normal human serum. All particles were washed and resuspended in 1 ml BSS.

### Isolation of neutrophils

Thioglycollate-elicited peritoneal neutrophils were collected from mice by peritoneal lavage 18 hours after intraperitoneal injection of 3% thioglycollate, and maintained in PBS with 5 U/ml heparin. The cells were centrifuged through Lymphoprep (density 1.077, Axis-Shield) at 400 g for 10 minutes and then washed and resuspended in BSS. Peripheral blood was obtained from mice by cardiac puncture, taken into heparin (5 U/ml) and immediately diluted with an equal volume of PBS with heparin (5 U/ml). This mixture was then diluted tenfold with PBS and one tenth volume of 10% dextran was added. After sedimentation for one hour the supernatant was removed, centrifuged through Lymphoprep (density 1.077) at 400 g for 10 minutes. The pellet was subjected to hypotonic lysis and the neutrophils pelleted at 200 g for 10 minutes. Cells were then resuspended in BSS. All experiments were performed on pooled blood from at least two mice. Human neutrophils were purified from peripheral blood by the standard procedures of dextran sedimentation, centrifugation through Lymphoprep and hypotonic lysis.

### Oxygen consumption

For the Seahorse apparatus (Seahorse Bioscience), 4×10^5^ peritoneal neutrophils (from 4–5 mice per experiment) in 100 μl BSS medium were added to the poly-L-lysine coated wells of 24 well microplates and incubated at room temperature for 30 minutes. 4×10^7^ opsonised, heat-killed *Candida* or 5 μM PMA were added to each well and oxygen consumption and extracellular pH were measured repeatedly using the Seahorse XFe96 Extracellular Flux Analyzer over 15 minutes. 5μM DPI was added to some wells immediately before the PMA.

The number of independent experiments and repeats are shown in Fig 2. For two of these experiments (shown in S6 Fig) the approximate number of cells per well at the end of each experiment (four separate measurements of PMA and *Candida* for WT and *Hvcn1* ^-/-^ each) were quantitated using the MTT assay [59] and normalised before averaging. These results were consistent with the previously obtained results (uncorrected for cell number) and thus all data were pooled.

Cell-Tak (BD Biosciences) 50ul of 22.4 ug/ml in PBS was added to each well of a 24 well microplate and incubated at room temperature for 30 minutes. Wells were washed twice with BSS and neutrophils, 5×10^5^ in 100ul BSS were added to each well and plates were centrifuged twice at 45g for 2 min. and incubated at 37°C for 30 min. Medium was changed for 600ul Assay buffer, RPMI (Sigma R8755) containing 5mM glucose, 2mM L-glutamine and 1mM sodium pyruvate (Assay buffer), 4×10^6^ heat killed Candida or PMA + DPI 5uM, were then added and measurements made in Seahorse XFe96 Extracellular Flux Analyzer over 45 min.

For Clarke type oxygen electrode (Rank Brothers) experiments, 1×10^7^ neutrophils were rapidly stirred in a at 37°C and1×10^8^ opsonised *Candida* were added and phagocytosis assessed microscopically.

### H_2_O_2_ generation

H_2_O_2_ was assayed with the Amplex Red Hydrogen Peroxide/Peroxidase Assay Kit (Invitrogen) in a 96 well plate with with 4×10^5^ neutrophils in 200ul/ well and 5uM PMA as stimulus.

### Confocal microscopy

Neutrophils (1–5×10^5^ from 2–3 mice per experiment in 300 μl BSS) were incubated for 30 minutes on 25 mm poly-L-lysine coated coverslips in Leiden dishes, or poly-L-lysine coated eight well ibi treated μ-Slides (Ibidi, Germany). For labelling of cytosol with SNARF, 1% by volume of 50 μg carboxy SNARF-1, AM ester, acetate in 100 μl DMSO was added for five minutes at room temperature. The cells were washed and resuspended in 400 μl BSS for the coverslip chamber and 200 μl for μ-Slides. For time course experiments, coverslips in Leiden chambers were incubated at 37°C on a heated stage and 2×10^6^ opsonised SNARF labelled organisms added. Images were taken every minute for 30–60 minutes. For end-point measurements in slide chambers the labelled cells were incubated for 15 minutes at 37°C and then 1×10^6^ particles were added and images obtained after a further 15–30 minutes at room temperature. All confocal experiments were performed on peripheral blood human or mouse neutrophils. Cells were imaged with a Zeiss 700 confocal microscope using a 63× oil immersion. SNARF-1 fluorescence was excited at 555 nm and the emission was split between two detectors measuring fluorescence emission simultaneously between 560–600 nm and *>*610 nm. Only cells containing a single *Candida* or latex particle were analysed.

### SNARF calibration

Although the pKa of SNARF in solution is ~7.5 at room temperature and 7.3–7.4 at 37°C, it has previously been described that the properties of the indicator change dramatically when exposed to cellular components such as proteins, carbohydrates and lipids. Soluble SNARF dye freely diffuses within a defined buffer system; bound SNARF may not be fully exposed to the surrounding solvent system and the response is thus modulated by the local environment. Also, the fluorescence response from some bound dye molecules may be polarised light (rotation of the dye is limited) and quenched (dye molecules bound near aromatic amino acids) [60,61] (and personal communication Thermo Fisher Scientific). Cytosolic pH standard curves were constructed by the nigericin/high K^+^ technique [17]. Cells were labelled with SNARF as described above and then washed and incubated in high K^+^ solutions (100 mM K^+^) containing 10 μM nigericin and 50 mM buffers at pH 3 (glycine), 4–6 (acetate), 7–9 (Tris), 10 (glycine) and 11–13 (PO_4_) in 100 mM KCl. Standard curves were also constructed with 100 mM KCl and a 50 mM mixture of barbital, citric acid, boric acid and PO_4_ adjusted to a pH of between 3 and 13. The standard curve in S4D Fig was buffered with Hepes. The cytoplasmic pH was allowed to equilibrate with each external solution until the ratio of SNARF no longer changed with time. In some experiments the cytoplasm was acidified by incubating the cells in 20mM NH4Cl (13% 150mM NH4Cl to 87% BSS) for 5 or 20 minutes. The medium was then replaced by BSS and the ratio of SNARF determined every minute for 15 minutes.

SNARF labelled *Candida* were equilibrated in the same buffers without nigericin. In addition, human neutrophils that had phagocytosed SNARF-labelled *Candida* were suspended in the buffers in the presence of 0.3% saponin before imaging the *Candida*.

SNARF-labelled *Candida* provide a suitable pH indicator system for measuring vacuolar pH

Standard curves in the two buffering systems demonstrated that the dynamic range of SNARF ranged between a pH of 6 and 11 (Fig 3D). The standard curve on intracellular organisms in permeabilised human neutrophils (Fig 3D) was not significantly different. There was considerable variation of the fluorescence ratio of the individual *Candida* organisms, particularly at higher pH. The fluorescent properties of the *Candida* were not permanently altered by the conditions in the vacuole. In some cases in *Hvcn1* ^-/-^ neutrophils (S3 Video and S4 Video) the vacuoles ruptured, releasing the organisms, the fluorescence of which reverted to levels comparable to those of other extracellular labelled organisms. Finally, there was no decrease in fluorescence intensity at the two emission wavelengths measured over the time course of the experiments (S7 Fig).

### Quantitation and statistical analyses

SNARF ratio (*>*610 nm / 560–600 nm) and vacuolar cross-sectional areas were determined by manual quantitation under blinded conditions using custom macro scripts in ImageJ (National Institutes of Health, USA, version *>*1.46r). Data were analysed using the R statistical package. For time courses, data were synchronised to the time of particle uptake and were normalised to the mean baseline pre-phagocytic ratio. Linear interpolation was performed between measurements. All statistical comparisons were undertaken by analysis of covariance (using the *lm* function) with experiment identifier as a covariate to correct for inter-experiment variation.

### pH dependence of myeloperoxidase peroxidatic, chlorinating catalatic and superoxide dismutase activities, and activities of leukocyte elastase and cathepsin G

MPO was purified from human neutrophils exactly as described previously [62]. Peroxidase and chlorination assays were conducted in 150mM NaCl containing 10 mM CHES, MES and HEPES at pH of 5–9 and 10, and also in MES (pH 5 and 6), potassium phosphate (pH 7 and 8) or Tris (pH 9 and 10), all at 50mM which were also used for catalatic and SOD measurements. Peroxidase activity was measured with 3,3′,5,5′-Tetramethylbenzidine (TMB) [63] in reactions containing 0.75 mM TMB, 300μM H_2_O_2_ and 4nM MPO with absorbance read at 655nm, and with 0-dianisidine (200 μM), 50mM H_2_O_2_ and 8nM MPO with absorbance read at 460nm. Chlorination was measured with monochlorodimedone (MCD, 20μM) [64], 100μM ethylenediaminetetraacetic acid, 8nM MPO, 100mM KCl and 50μM H_2_O_2_ and read at 290nm. Catalatic activity of MPO was determined using a Hansatech Oxygraph oxygen electrode to measure the initial rate of oxygen release after addition of hydrogen peroxide. 1 mL of buffer was partially deoxygenated with nitrogen gas before addition of 1.5 μM MPO and assembly of the chamber top. The reaction was initiated by addition of 12.5–500μM H_2_O_2_. Relative activities of the SOD capability of MPO were assessed from the rate of nitroblue tetrazolium (NBT) reduction by xanthine oxidase-generated superoxide and its inhibition by MPO, using a method adapted from Kettle and Winterbourn [65]. Buffer containing 100μM methionine, 100μM NBT and 50μM hypoxanthine was placed in a cuvette and NBT reduction rate after addition of xanthine oxidase (XO) was monitored at 560 nm. An absorption coefficient of 15 mM^−1^ cm^−1^ was used for the formazan product at 560 nm [66]. The amount of XO (3.3 μg/mL at pH 8) was varied at different pH values so that the rate of NBT reduction was the same at all pH values. The inhibitory effect of 40–100 nM MPO was measured to assess its relative superoxide dismutase activity at different pH values.

Elastase (5mU/ml) was incubated with 10μM N-Methoxysuccinyl-Ala-Ala-Pro-Val-7-amido-4-methylcoumarin at 37°C Excitation 355nm Emission 460nm. Substrate for cathepsin G (25mU/ml) was 2mM N-Succinyl-Ala-Ala-Pro-Phe p-nitroanilide at 37°C with absorbance measured at 410nm.

### Mice

The knock-out mouse strains used in this study were: *Hvcn1* ^-/-^[17], and X-CGD mice B6.129S6-Cybbtm1Din/J deficient in gp91^phox^ (The Jackson Laboratory). Mice were genotyped according to previously described protocols.

## Supporting Information

S1 Fig Titration of the buffering capacity of *Candida* on pH changes in response to the addition of KOH. Two independent experiments (red and black) are shown.
(PDF)

S2 Fig Spectral changes induced by the binding of different concentrations of cyanide to myeloperoxidase in intact human neutrophils. Intact neutrophils (1.5×10^7^ in BSS) in a 1 cm pathlength cuvette were placed in an in-house built scanning spectrometer equipped for analyses of turbid materials and a baseline was recorded. Cyanide was added at the concentrations shown and the resulting difference spectra were recorded after the optical change had stabilised (within a few minutes). The resulting difference spectra exhibit features at 419(-), 461(+), 575(-) and 639(+) nm that are typical of the formation of the ferric MPO-cyanide complex. Using an extinction coefficient for cyanide binding at 461–419 nm of 68 mM^-1^cm^-1^ (estimated from data in [67]) the concentration of MPO in the cell suspension was 0.45μM.
(PDF)

S3 Fig The lack of an effect of 5mM sodium azide of pH dependence of SNARF ratio. SNARF labelled *Candida* were incubated in the Barbital buffer at pH of 7, 9, 10 and 11 for 30 min. and the fluorescence measured. The numbers of measurements are given at the bottom of each bar. Mean ± SD are shown.
(PDF)

S4 Fig Rates of recovery of cytoplasmic pH in *Hvcn1* ^-/-^ and wild type mouse neutrophils after incubation for five (A) or 20 minutes (B) with 20mM NH_4_Cl followed by its replacement by BSS. The SNARF ratios in each cell were measured every minute and are expressed as the mean ± SD, the numbers of cells studied are shown. The standard curve of SNARF ratio against pH is shown in (C) and the cytosolic ratios and corresponding pH of 470 and 480 cells from eight experiments on WT and *Hvcn1* ^-/-^ cells respectively are shown in (D).
(PDF)

S5 Fig pH dependence of superoxide dismutase and catalatic activities of myeloperoxidase. Results are shown as a percentage of maximal activity. (PDF)

S6 Fig Oxygen consumption (OCR, A) and extracellular acidification rate (ECAR, B) for two experiments normalised for cell number using the MTT assay. Data are shown in arbitrary units post MTT correction. For *PMA* and *Candida* in both WT and *Hvcn1* ^-/-^, four separate measurements were taken in each of the two independent experiments.
(PDF)

S7 Fig Demonstration of the time course of changes in intensity of the two emission wavelengths from the experiment shown in Fig 4B showing that there is no loss of intensity over time. (PDF)

S1 Video Time lapse video of human neutrophils phagocytosing *Candida*. (WMV)

S2 Video Time lapse video of human neutrophils phagocytosing *Candida* with the addition of 5 μM DPI during the time course. (WMV)

S3 Video Time lapse video of *Hvcn1* ^-/-^ neutrophils phagocytosing *Candida*. Following uptake the Candida particles turn red indicating extreme alkalinisation and the vacuoles can be seen to swell. In a small number of cells the cytoplasm suddenly becomes alkaline.
(WMV)

S4 Video Time lapse video of an *Hvcn1* ^-/-^ neutrophil phagocytosing and releasing a *Candida* particle which is subsequently phagocytosed by an adjacent neutrophil. (WMV)
