# Supplementary figures and images for: Alkalinity of Neutrophil Phagocytic Vacuoles Is Modulated by HVCN1 and Has Consequences for Myeloperoxidase Activity
Source: PLoS One. 2015 Apr 17;10(4):e0125906. doi: 10.1371/journal.pone.0125906 (PMC4401748; doi:10.1371/journal.pone.0125906)

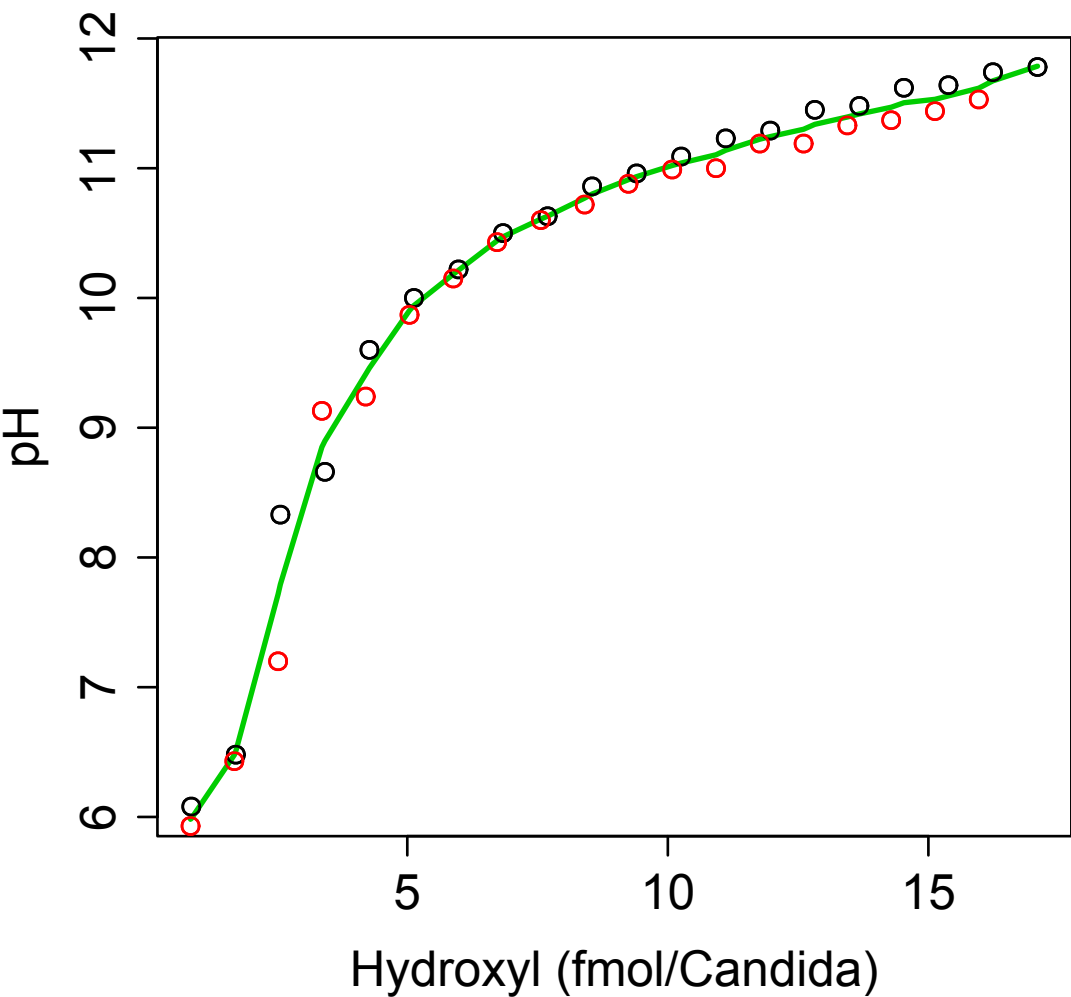

Supplement: S1 Fig — Two independent experiments (red and black) are shown. (PDF) [file pone.0125906.s001.pdf]

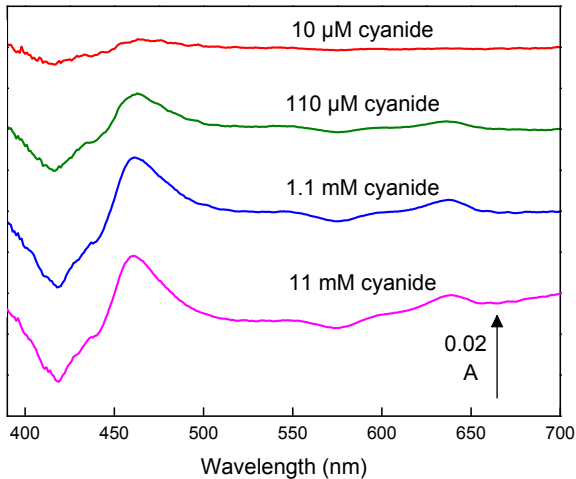

Supplement: S2 Fig — Intact neutrophils (1.5×10^7^ in BSS) in a 1 cm pathlength cuvette were placed in an in-house built scanning spectrometer equipped for analyses of turbid materials and a baseline was recorded. Cyanide was added at the concentrations shown and the resulting difference spectra were recorded after the optical change had stabilised (within a few minutes). The resulting difference spectra exhibit features at 419(-), 461(+), 575(-) and 639(+) nm that are typical of the formation of the ferric MPO-cyanide complex. Using an extinction coefficient for cyanide binding at 461–419 nm of 68 mM-1cm-1 (estimated from data in [67]) the concentration of MPO in the cell suspension was 0.45μM. (PDF) [file pone.0125906.s002.pdf]

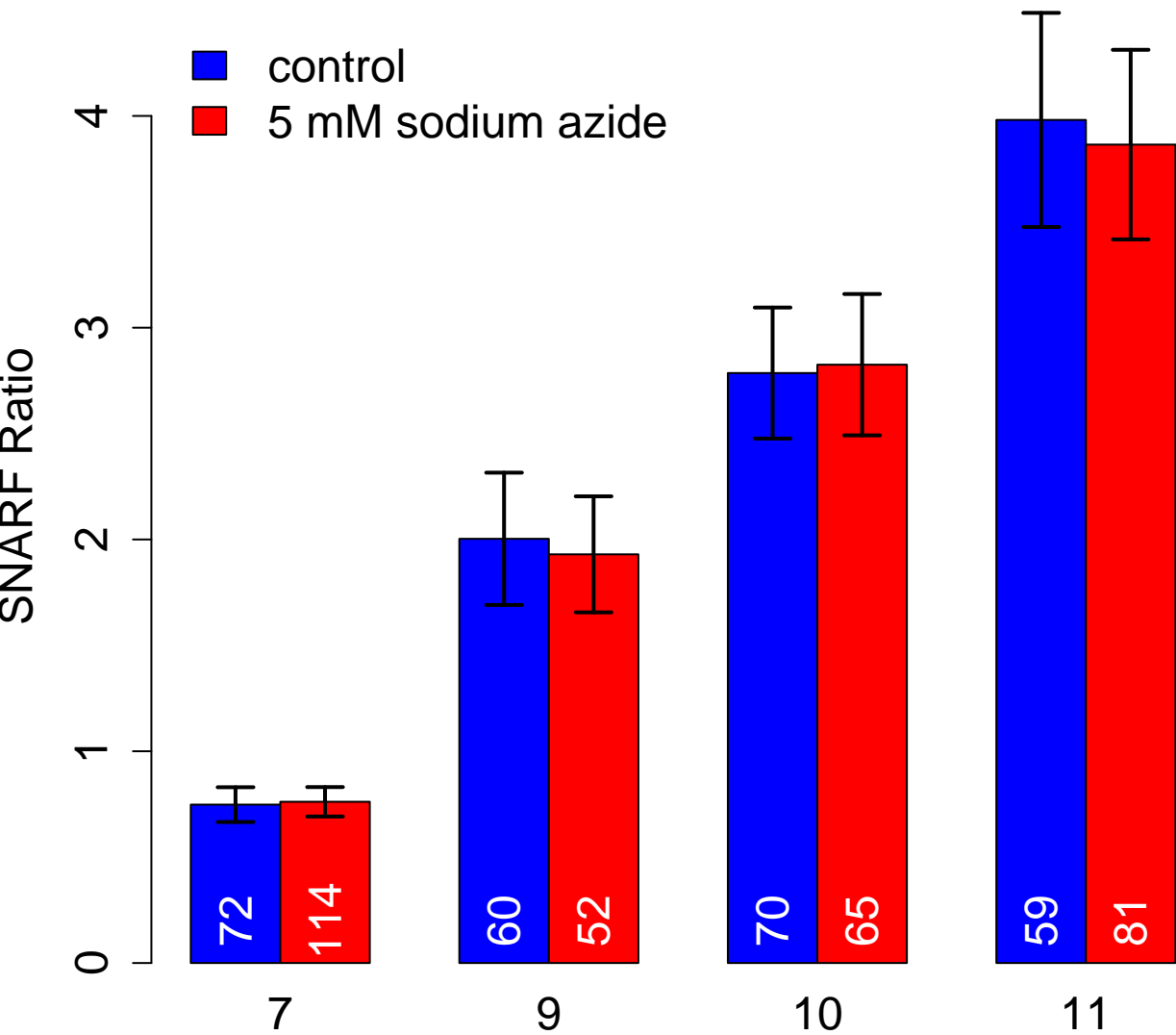

Supplement: S3 Fig — SNARF labelled Candida were incubated in the Barbital buffer at pH of 7, 9, 10 and 11 for 30 min. and the fluorescence measured. The numbers of measurements are given at the bottom of each bar. Mean ± SD are shown. (PDF) [file pone.0125906.s003.pdf]

■ HVCN1<sup>-/-</sup> ■ WT

SNARF Ratio

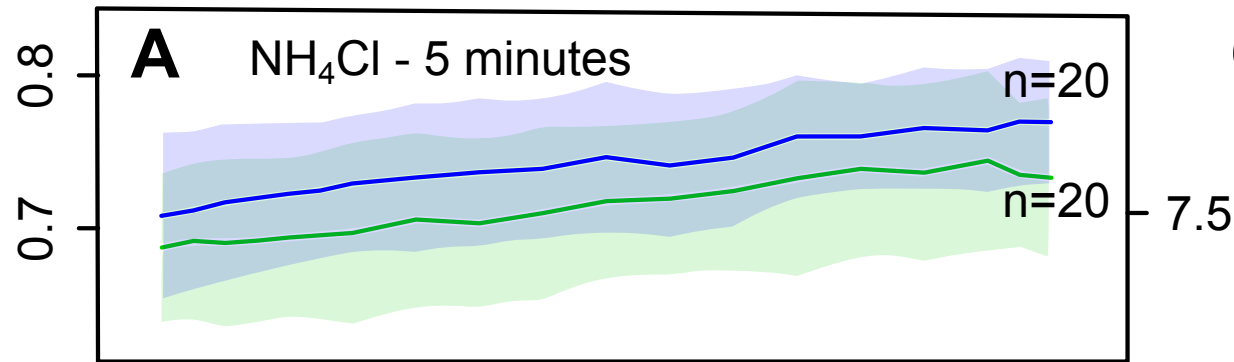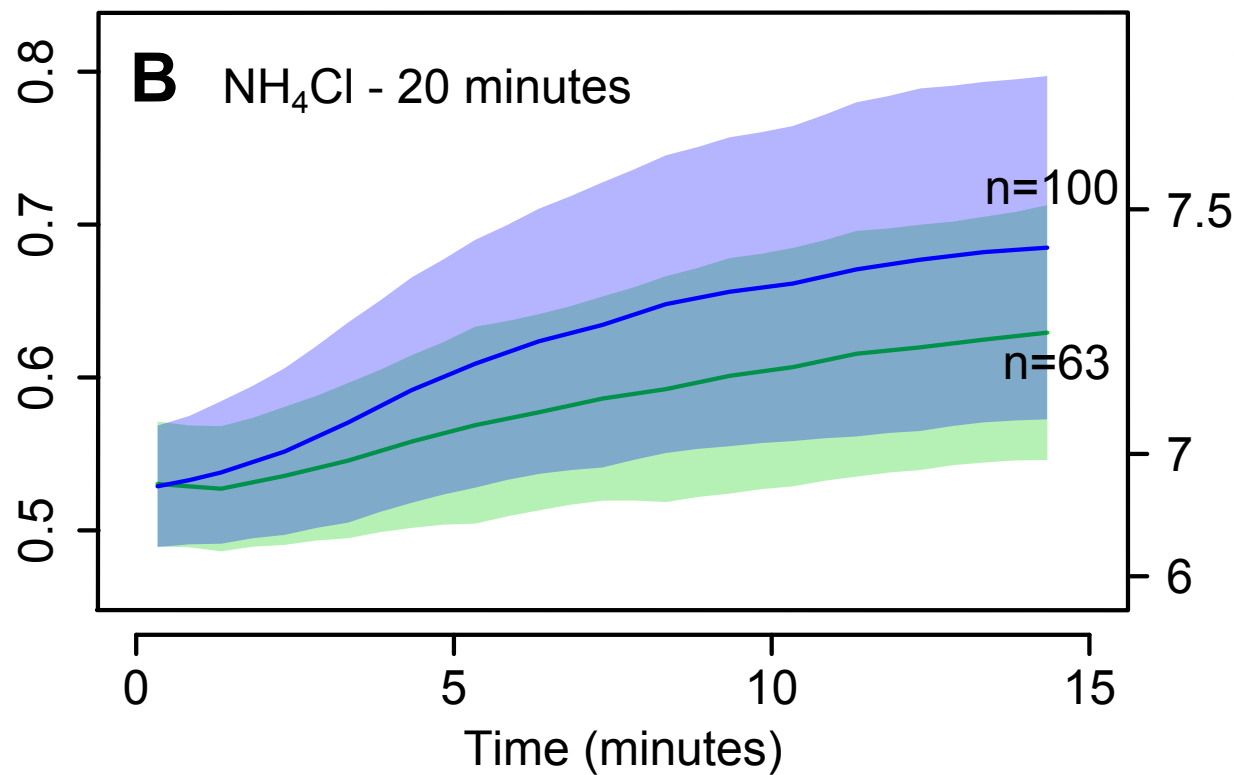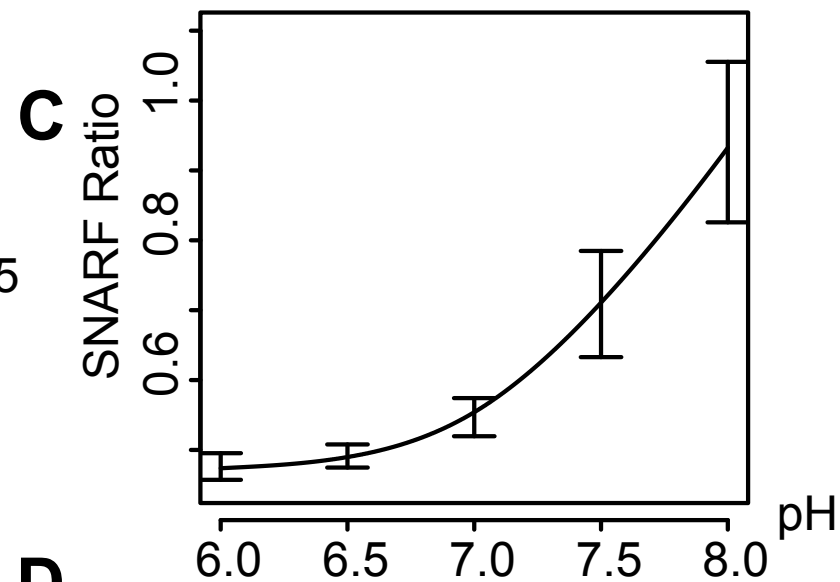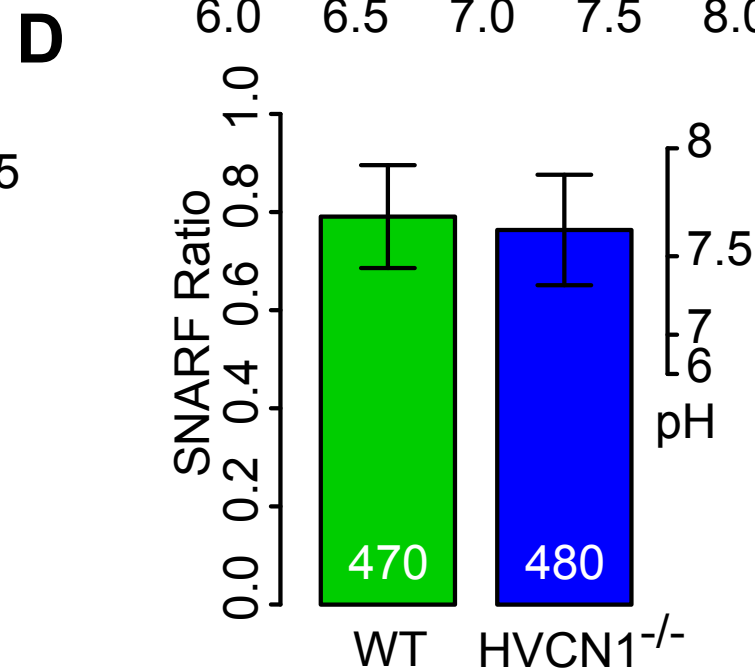

Supplement: S4 Fig — The SNARF ratios in each cell were measured every minute and are expressed as the mean ± SD, the numbers of cells studied are shown. The standard curve of SNARF ratio against pH is shown in (C) and the cytosolic ratios and corresponding pH of 470 and 480 cells from eight experiments on WT and Hvcn1 -/- cells respectively are shown in (D). (PDF) [file pone.0125906.s004.pdf]

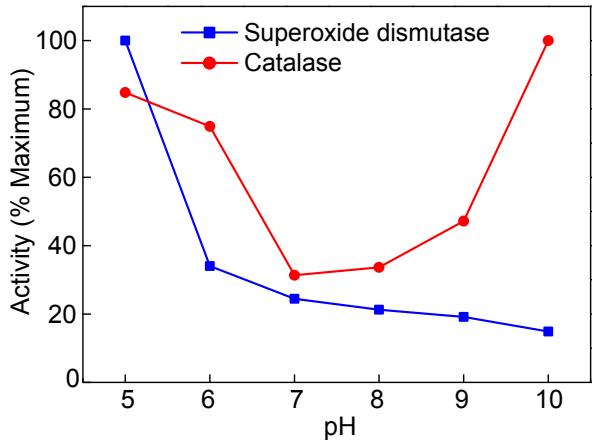

Supplement: S5 Fig — (PDF) [file pone.0125906.s005.pdf]

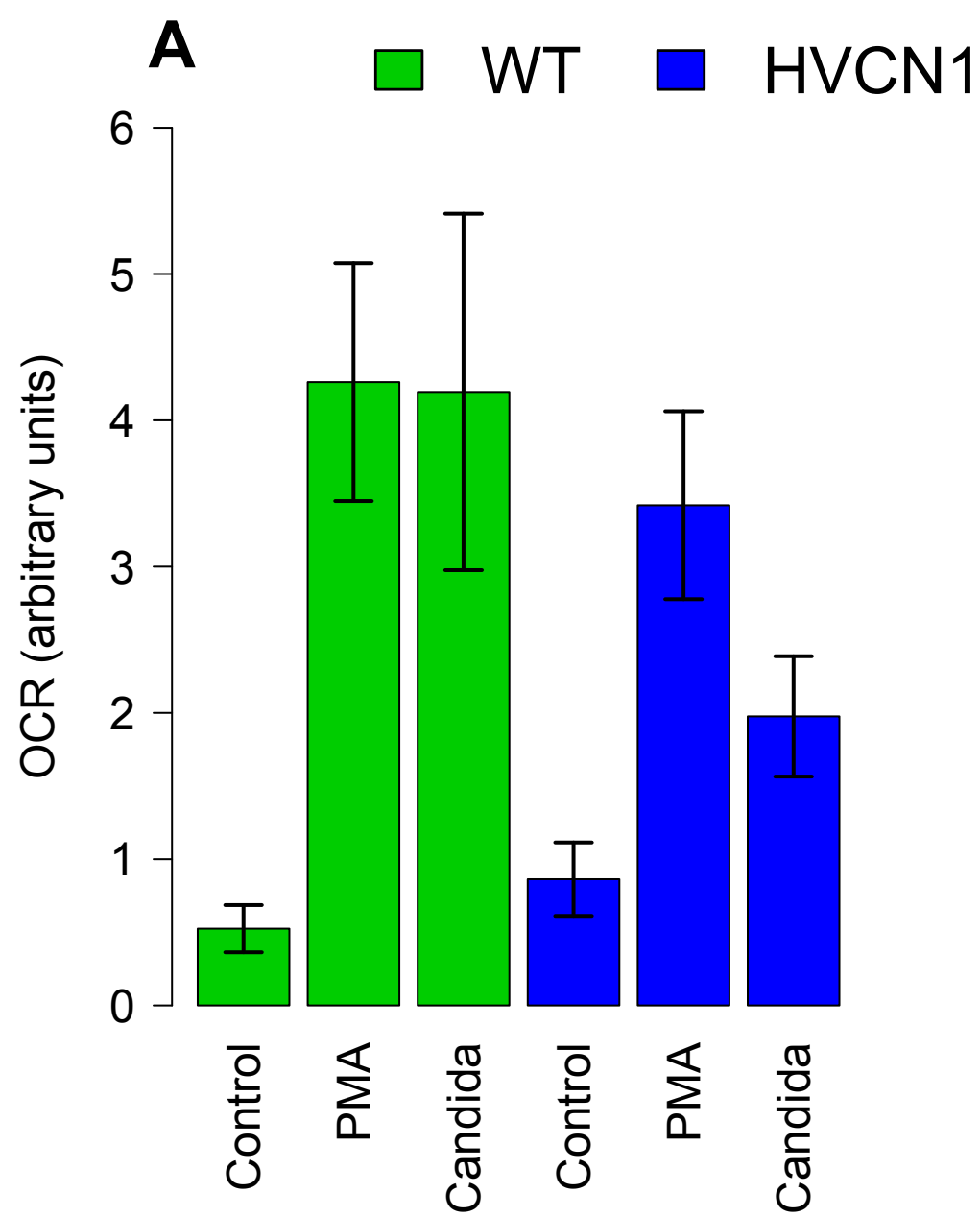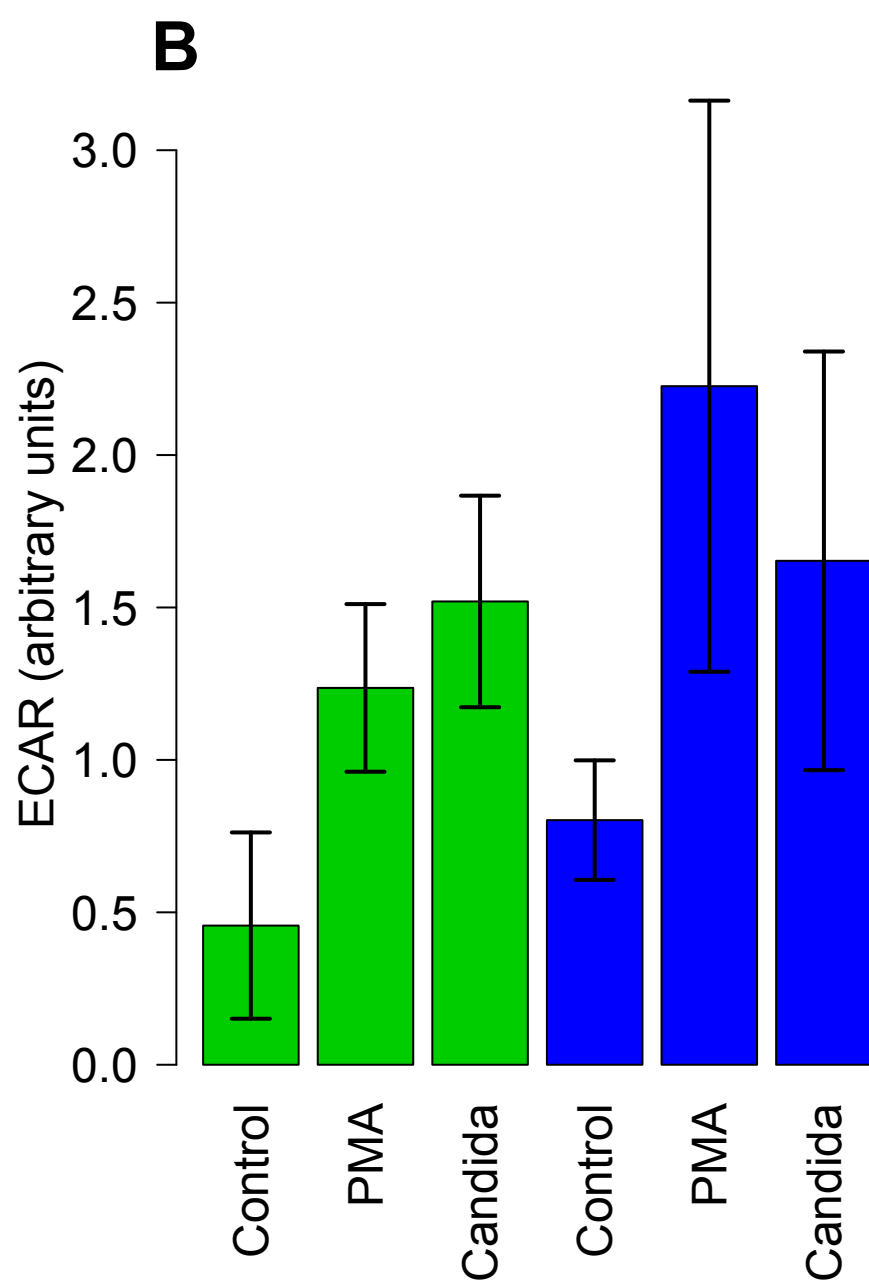

Supplement: S6 Fig — Data are shown in arbitrary units post MTT correction. For PMA and Candida in both WT and Hvcn1 -/-, four separate measurements were taken in each of the two independent experiments. (PDF) [file pone.0125906.s006.pdf]

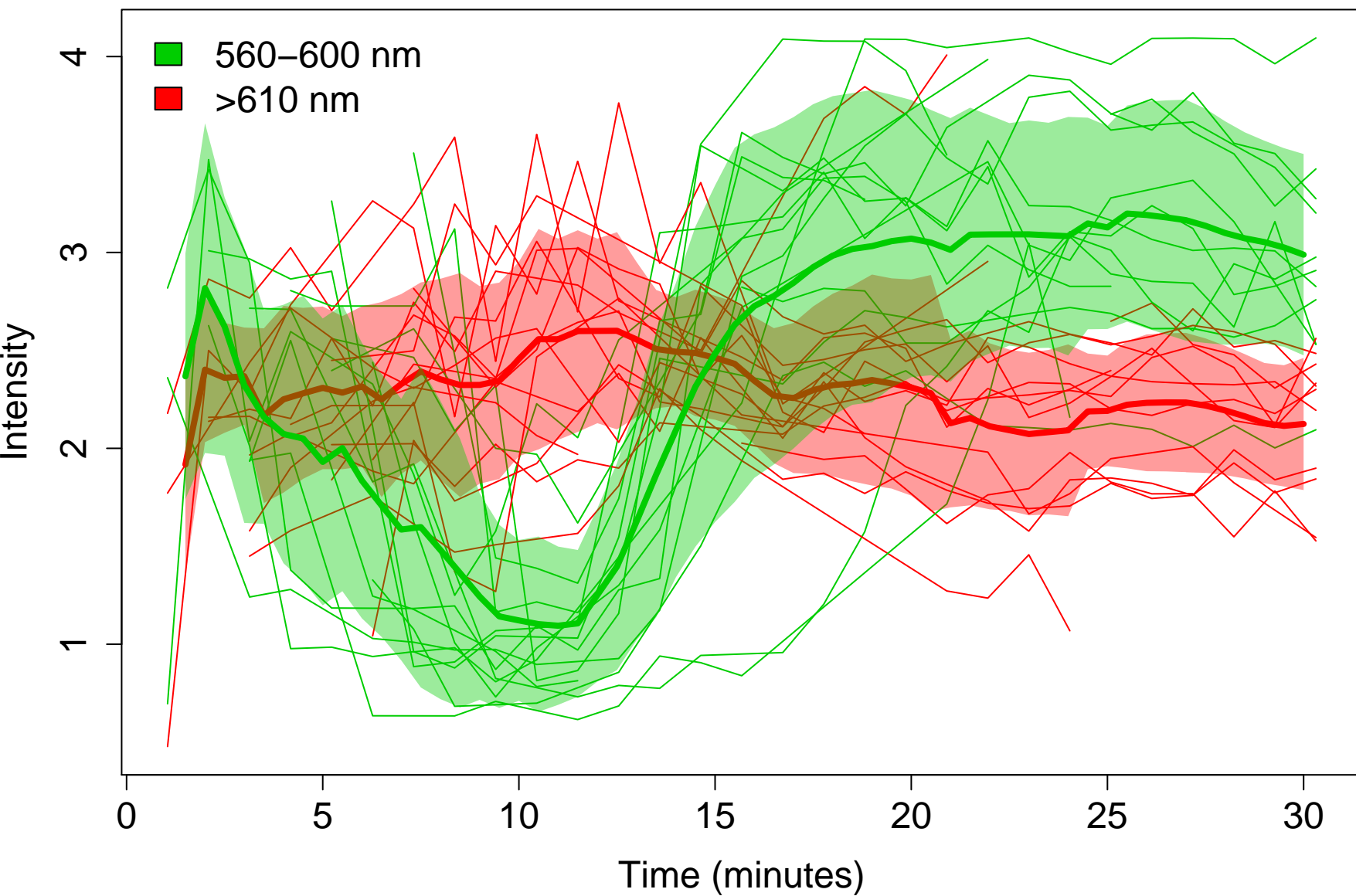

Supplement: S7 Fig — (PDF) [file pone.0125906.s007.pdf]
